# The Influence of Maternal Islet Beta-Cell Autoantibodies in Conjunction with Gestational Hyperglycemia on Neonatal Outcomes

**DOI:** 10.1371/journal.pone.0120414

**Published:** 2015-03-18

**Authors:** Li Zhe, Wu Tian-mei, Ming Wei-jie, Chen Xin, Xiao Xiao-min

**Affiliations:** The Department of Obstetrics and Gynecology at the 1^st^ Affiliated Hospital of Jinan University, Guangzhou, China; University of Texas Health Science Center at San Antonio, UNITED STATES

## Abstract

**Objective:**

To determine the predictive value of the presence of maternal islet beta-cell autoantibodies with respect to neonatal outcomes.

**Methods:**

A total of 311 pregnant women with abnormal 75 g oral glucose tolerance test (OGTT) results were enrolled in this study. Maternal glutamic acid decarboxylase autoantibodies (GADA), islet cell autoantibodies (ICA) and insulin autoantibodies (IAA) were tested in fasting blood both on the day following the routine OGTT and before delivery. The birth weight, Apgar score, blood glucose and outcomes of each neonate were later evaluated and recorded.

**Results:**

1. In this study, 33.9% of the pregnant women with gestational hyperglycemia had detectable levels of one or more types of anti-islet cell antibodies in the third trimester. The proportion of women who produced GADA and/or ICA was significantly higher in the group of women with gestational hyperglycemia than in the control group (*P<0*.*05*). The groups similarly differed in the proportion of women who tested positive for any anti-islet cell antibody (*P*<0.05). 2. Of the patients in our study, those who produced GADA exhibited an increase in uterine and umbilical arterial pulsatility indexes (PIs) during the third trimesters compared with the control group (*P*˂0.05). Additionally, an increased frequency of fetal growth restriction (FGR) was observed in the infants of women who produced IAA during pregnancy compared with those without autoantibodies (*P*˂0.05). 3. The rate of newborn admission to the neonatal intensive care unit (NICU) was significantly associated with the presence of maternal ICA during the third trimester (OR, 6.36; 95% CI, 1.22–33.26). 4. The incidence of neonatal asphyxia was associated with the presence of maternal GADA in both the second (OR, 10.44; 95% CI, 1.46–74.92) and the third (OR, 8.33; 95% CI, 1.45–47.82) trimesters.

**Conclusion:**

Approximately one-third of the women with gestational hyperglycemia produced anti-islet cell antibodies. The incidence of FGR was higher in women with gestational hyperglycemia who produced IAA than in those without autoantibodies. Maternal ICA production in the third trimester was a risk factor for the subsequent admission of newborns to the NICU. Furthermore, the presence of maternal GADA placed the neonate at increased risk for asphyxia.

## Background

The major mechanism underlying gestational diabetes mellitus (GDM) has long been thought to be insulin resistance. However, recent work suggests that patients with GDM may actually exhibit autoimmunity against islet cells [1]. In our previous publications, antibodies against beta-cells and insulin were detected during the second trimester in approximately one-third of women with gestational hyperglycemia [2]. These findings revealed that certain women with gestational hyperglycemia might also qualify as having subclinical type 1 diabetes.

Islet beta-cell autoantibodies primarily consist of glutamic acid decarboxylase autoantibodies (GADA) [3–5], islet cell autoantibodies (ICA) [6], insulin autoantibodies (IAA) [7], tyrosine phosphatase-like protein antibodies (IA-2A) and ZnT8 autoantibodies (ZnT8A). Screening for these autoantibodies has been performed in women with GDM for the early detection of type 1 diabetes [8]; however, few reports have examined the impact of the presence of these autoantibodies during gestation on the resulting neonates. Therefore, this study evaluated the prevalence of anti-islet cell antibodies in women with gestational hyperglycemia and the relationship between the presence of maternal islet beta-cell autoantibodies and neonatal outcomes to further inform and guide future clinical care for these women and their neonates.

## Materials and Methods

### Data Collection

This study was approved by the 1^st^ Affiliated Hospital of Jinan University in China. Routine oral glucose tolerance test (OGTT) data were collected at this hospital from March 2010 through March 2011. The 311 cases of interest were chosen after an abnormal 75 g OGTT result (i.e., the fasting, 1-h or 2-h plasma glucose (PG) values exceeded 5.6, 10.3 or 8.6 mmol/L, respectively), and informed consent was obtained from the patients. Informed consent was also obtained from an additional 60 pregnant women with normal 75 g OGTT results (with fasting, 1-h and 2-h PG values below 5.1, 10.0, and 8.5 mmol/L, respectively), who were included in the study to serve as a control cohort. All of the included patients were Chinese women in their first pregnancy and had no previous history of diabetes, hyperthyroidism or autoimmune disease and no prior blood transfusions, transplants or immunotherapies.

### Prenatal Tests and Guidelines

Pregnant women with abnormal glycometabolism and control pregnant women were subjected to fasting blood tests for the presence of GADA, ICA and IAA both on the day following the routine OGTT and before delivery. Following their abnormal OGTT result, all hyperglycemic patients received diet, exercise and blood glucose monitoring guidelines. The PIs of both the uterine and the umbilical arteries in the third trimester were recorded for all women enrolled in this study. If either PI exceeded the 95^th^ percentile, it was considered to be abnormal.

### Neonatal Data Collection

The data ultimately recorded for the neonates of the enrolled pregnant women consisted of birth weight, Apgar score and blood glucose levels as well as the presence of any malformations, neonatal respiratory distress syndrome (NRDS) or other diseases.

### Measurement of Beta-Cell-Specific Autoantibodies

GADA were measured by radioimmunoassay using a kit obtained from MEDIPAN Inc., Germany. The serum samples were assayed for ICA and IAA using ICA and IAA enzyme-linked immunosorbent assay (ELISA) kits, respectively, from BIOMEARIC, United States, according to the manufacturer’s instructions.

### Data Analysis

All data are presented as the mean ± standard error. Fetal outcomes were analyzed by rank sum tests, and a value of *P*<0.05 was considered statistically significant. Factors related to the neonatal intensive care unit (NICU) and neonatal asphyxia were analyzed by stepwise forward multiple and univariate logistic regressions, respectively, with values of *P*<0.1 considered statistically significant. All data were analyzed with SPSS 13.0 statistical software (SPSS, Inc., Chicago, IL, USA).

### Ethics

This study was approved by the Ethics Committee of the 1^st^ Affiliated Hospital of Jinan University. All participants, including the enrolled pregnant women and their next of kin, caretakers and/or guardians, provided written informed consent to participate in this study.

## Results

### 1. The frequency of beta-cell autoantibodies in women with gestational hyperglycemia in their third trimester

The presence of at least one type of autoantibody (ICA, GADA and/or IAA) was observed in 84 of 248 (33.9%) women with gestational hyperglycemia in their third trimester (Table 1). Specifically, 5.2%, 29.0%, 3.6% and 8.5% of the women tested positive for GADA, IAA, ICA, or a combination of GADA and ICA, respectively. The proportion of women with gestational hyperglycemia producing GADA and/or ICA and the proportion who were positive for at least one type of tested autoantibody were both significantly higher than in the control group (*P*<0.05).

**Table 1:** The frequency of maternal beta-cell autoantibody production in the third trimester.

	GADA+	IAA+	ICA+	GADA+ and/or ICA+	At least one antibody+
Study group (n = 248)	13 (5.2)	72 (29.0)	9 (3.6)	21 (8.5)	84 (33.9)
Control group (n = 60)	0 (0)	10 (16.7)	0 (0)	0 (0)	10 (16.7)
*P* value	0.146	0.052	0.284	0.040	0.009

### 2. The impact of maternal islet beta-cell autoantibodies on neonatal outcomes

Patients producing GADA exhibited an increase in uterine and umbilical arterial PIs during the third trimester compared with the control group (Table 2, *P*<0.05). Additionally, the fetuses of patients with IAA were more likely to experience fetal growth restriction (FGR) (*P*<0.05). In contrast, the incidence of fetal malformation and neonatal asphyxia was higher in neonates from women with GADA; however, there was no significant difference between those producing GADA and those without autoantibodies (0.05≤*P*≤0.15). Additionally, fetuses from pregnant women producing ICA showed higher rates of fetal malformation, although there was no significant difference compared with the non-antibody-producing group (0.05≤*P*≤0.15).

**Table 2:** Fetal outcomes in the autoantibody-producing and non-autoantibody-producing groups and the control group.

Index	GADA+ (N = 16)	ICA+ (N = 10)	IAA+ (N = 89)	Antibody− (N = 183)	Control group (N = 60)
PIs of uterine arteries and umbilical arteries increased during the third trimester (%)	28.6^II^	10.0	16.0^III^	15.8^III^	5.7
FGR (%)	6.3	10.0	7.9 ^B^	1.6	1.7
Fetal malformation (%)	6.3^C^	10.0^C^	1.1	0	1.7
Stillbirth (%)	0	0	0	0.5	0
Gestational age (%)	274.5±10.3	276.7±7.0	273.1±11.4	273.8±10.4	NA
Premature delivery (%)	6.3	0	4.5	8.2^II^	0
Neonate birth weight (%)	3090±471	3195±547	3144±513	3215±413	3172±344
Low birth weight (%)	12.5	10.0	9.0^D^	3.8	1.7
Macrosomia (%)	0	0	5.6	3.8	0
Amniotic fluid abnormalities (%)	18.8	20.0	15.7	16.4	20.0
Moderate to severe meconium-stained liquor (%)	0	10.0	5.6^III^	10.4	15.0
Neonatal asphyxia (%)	12.5^D^	0	2.2	2.7	1.7
Rate of admission to the NICU (%)	56.3^II^	70.0^I^	42.7^I^	42.9^I^	20.0
Adverse pregnancy outcomes	6.3	0	1.1	0.5	0

Compared with the non-antibody-producing group: A: *P*<0.01, B: 0.01≤*P*<0.05, C: 0.05≤*P*<0.10, and D: 0.10≤*P*<0.15.

Compared with the control group: I: *P*<0.01, II: 0.01≤*P*<0.05, and III: 0.05≤*P*<0.10.

### 3. Multiple logistic regression analysis

Data were collected from each case, and logistic regression analysis was subsequently performed for several variables, including uterine and umbilical arterial PIs during the third trimester; the gestational age at delivery; the amniotic fluid index; the characteristics of the amniotic fluid; and the incidence of spontaneous abortion, premature delivery, neonatal malformation, stillbirth, hypertensive disorder, pregnancy complications, intrauterine growth restriction, premature rupture of the fetal membranes, placental abruption, soft birth canal laceration, postpartum hemorrhage and caesarean section. The neonatal birth weight, the Apgar score, the blood glucose level, and the presence of NRDS or other diseases were also noted following birth.

**3.1 Factors Contributing to Neonatal Admission to the NICU.** Stepwise forward multiple logistic regression analysis was performed to examine the effect of independent variables on neonatal admission to the NICU. Candidate variables were included in the model if their *P* value was less than 0.05 when compared with the control group in the univariate analysis. Risk factors for neonatal admission to the NICU included premature delivery (OR, 11.08; 95% CI, 2.28–53.73), the characteristics of the amniotic fluid (OR, 3.23; 95% CI, 1.82–5.73), the OGTT 1-h plasma glucose (PG) result (OR, 1.28; 95% CI, 1.04–1.59), and the presence of maternal ICA in the third trimester (OR, 6.36; 95% CI, 1.22–33.26) (Table 3).

**Table 3:** Multiple logistic regression analysis of the NICU admission rate.

Relevant factor	OR (95% CI)	*P*
Premature delivery	11.08 (2.28–53.73)	0.003
Characteristics of amniotic fluid	3.23 (1.82–5.73)	0.000
OGTT 1-h PG result	1.28 (1.04–1.59)	0.020
ICA-positive in the third trimester	6.36 (1.22–33.26)	0.029

**3.2 Factors Contributing to Neonatal Asphyxia.** Based on the univariate logistic regression analysis, low birth weight, the presence of maternal GADA in the second trimester, and the presence of maternal GADA in the third trimester were risk factors for neonatal asphyxia, with ORs of 19.25 (95% CI, 3.74–99.08), 10.44 (95% CI, 1.46–74.92) and 8.33 (95% CI, 1.45–47.82), respectively (Table 4).

**Table 4:** Univariate logistic regression analysis of neonatal asphyxia.

Relevant factor	OR (95% CI)	*P*
Low birth weight	19.25 (3.74–99.08)	0.003
GADA-positive in the second trimester	10.44 (1.46–74.92)	0.046
GADA-positive in the third trimester	8.33 (1.45–47.82)	0.046

## Discussion

### The frequency of beta-cell autoantibody production in women with gestational hyperglycemia in their third trimester

1. In this study, 33.9% of women with gestational hyperglycemia produced at least one type of anti-islet cell antibody during their third trimester. Additionally, the proportion of women with gestational hyperglycemia producing GADA and/or ICA and the proportion who were positive for at least one type of tested autoantibody were both significantly higher than the proportions in the control group (*P*<0.05). Several studies [9–12] have concluded that pregnant women who produce anti-islet cell antibodies have an increased risk of developing diabetes mellitus. Furthermore, Fuchtenbusch [13] demonstrated that at least 29% of women who had produced autoantibodies during pregnancy and 84% of women who had produced all three types of autoantibodies (GADA, ICA and IAA) during pregnancy exhibited symptoms of type 1 diabetes mellitus (T1DM) two years after delivery. However, pregnancies with autoantibody production and gestational hyperglycemia have not yet been systematically evaluated. Wucher [14] found that in 21 pregnant women with GDM who developed T1DM after delivery, only 8 of these patients had been diagnosed with gestational hyperglycemia and concurrent production of abnormal autoantibodies.

### 2. The influence of maternal islet beta-cell autoantibodies with concurrent gestational hyperglycemia on neonatal outcomes

Our study found that neonates from IAA-producing patients exhibited a higher incidence of FGR than did neonates from patients who did not produce these antibodies (*P*<0.05). Univariate logistic regression analysis also suggested an increased risk of neonatal asphyxia when the neonate had a low birth weight or when maternal GADA were produced in the second or third trimester, with ORs of 19.25 (95% CI, 3.74–99.08), 10.44 (95% CI, 1.46–74.92) and 8.33 (95% CI, 1.45–47.82), respectively. Moreover, multiple logistic regression analysis suggested that ICA production in late pregnancy is a risk factor for neonatal admission to the NICU (OR, 6.36, 95% CI, 1.22–33.26). Due to the increased risk of FGR, close attention should be paid to maternal cases of GDM when IAA are also produced. Additionally, in our study, GADA production and ICA production were risk factors for neonatal asphyxia and neonatal admission to the NICU, respectively. Taken together, these results suggest that the presence of maternal autoantibodies against beta-cell antigens is associated with poorer neonatal outcomes. However, the mechanisms underlying the associations between these autoantibodies and gestational outcomes remain unclear. In our study, a higher percentage of patients showed increased uterine and umbilical arterial PIs among those patients producing GADA during the third trimester than among those in the control group (*P*<0.05), suggesting that the presence of autoantibodies against beta-cell antigens may influence the uterine placental vasculature. This effect on the placental vasculature, combined with the high resistance of the uterine and umbilical arteries, may result in placental insufficiency. It is important to note that previous work [15–17] has shown that both mothers with GDM and mothers with T1DM exhibit a significant increase in total lymphocytes, whereas their newborns exhibit a reduction in the number of natural killer lymphocytes. Additionally, Holm [18] found an association between autoantibody production and increases in inflammatory factors, such as interleukin-1β. Therefore, the inflammatory response and immunoreaction may influence the ability of the placenta and cord to function, leading to adverse pregnancy outcomes. However, Holm also showed that GADA levels in cord blood plasma correlated positively with the percentage of CD4^+^CD25^+^ T cells and CCR4 expression in these CD4^+^CD25^+^ T cells. Therefore, elucidation of the exact underlying mechanism requires further investigation. Li [19] and Yuan [20] demonstrated that patients with GDM who produced antibodies against beta-cell antigens had a higher risk of pregnancy failure than those lacking antibodies against beta-cell antigens did, including pregnancies that resulted in overweight or low-birth-weight newborns. However, their study included spontaneous abortions, stillbirths, fetal malformations, premature deliveries and FGR as pregnancy failures. By contrast, our study did not include premature deliveries or FGR as pregnancy failures, which may explain why there was no significant difference between the hyperglycemic cohort and the control cohort in terms of pregnancy failures, macrosomia or low neonatal birth weight. Additionally, Wucher [9] reviewed 21 cases of women who were diagnosed as diabetic during a pregnancy (“index pregnancy”) who then progressed to T1DM after delivery and were also producing GADA and/or IA-2A. Abnormal outcomes occurred in 14 of the 21 pregnancies, including two fetal deaths, four preterm deliveries and eight macrosomic infants, although no congenital malformations were reported. Buschard [21] found that obstetric complications, such as toxemia, occurred in 9.5% of patients who developed insulin-dependent diabetes mellitus during pregnancy and that these patients had a perinatal mortality rate of 6.3%. Both of these rates are higher than those in the general population but similar to those of patients with T1DM who were diagnosed prior to pregnancy. In contrast, the frequency of malformations in his study was 1.6%, which is the same as that in the general population (1.4%) but lower than that observed in patients with long-standing diabetes. This finding suggests that hyperglycemia manifests in the second trimester. Based on the two cited studies, we hypothesize that the fetal prognosis in cases of autoimmune GDM is worse than in cases of GDM with an absence of autoantibodies. However, after analyzing the inclusion criteria and evaluating the enrolled patients based on the guidelines of the International Association of Diabetes and Pregnancy Study Groups (IADPSG) [22], we determined that the participants in these two studies should have been diagnosed with overt diabetes mellitus due to their high levels of fasting sugar, random blood glucose and HbA1c.

In conclusion, a significant fraction of pregnant women with gestational hyperglycemia produce autoantibodies. Therefore, we should be highly attentive to the possible development of T1DM. Furthermore, the influence of maternal autoantibodies on the neonate should be an area of focus. In particular, uterine and umbilical arterial PIs in patients producing autoantibodies, as well as the condition of the fetus, should be closely monitored during gestation to avoid NRDS and other diseases.

One limitation of our study was that the criteria used to assess the OGTT results in our study group were instituted by the Chinese Society of Perinatal Medicine and therefore are Chinese criteria, rather than IADPSG criteria. The criteria used in our study, the American Diabetes Association (ADA) criteria and the IADPSG criteria for normal glucose tolerance (NGT) and gestational diabetes are listed in Table 5. This table indicates that after the IADPSG and ADA revised the criteria for the OGTT in 2012, the control group in our study did not include patients with GDM. On the other hand, based on the Chinese criteria, our study group did include patients who met the IADPSG/ADA criteria. Another major limitation of this study was that the cohorts were ultimately not large enough to obtain a significant difference. Therefore, further investigation is currently underway with a larger sample size.

**Table 5:** The 75 g OGTT criteria used in this study and the ADA and IADPSG criteria for differentiating NGT and gestational diabetes.

	NGT	GDM
This study	Fasting: ≤5.1 mmol/L; 1 h: ≤10.0 mmol/L; 2 h: ≤8.5 mmol/L	Any of the following PG values are exceeded: fasting: ≥5.6 mmol/L; 1 h: ≥10.3 mmol/L; 2 h: ≥8.6 mmol/L
ADA	Fasting: ≤5.1 mmol/L; 1 h: ≤10.0 mmol/L; 2 h: ≤8.5 mmol/L	Any of the following PG values are exceeded: fasting: ≥5.1 mmol/L; 1 h: ≥10.0 mmol/L; 2 h: ≥8.5 mmol/L
IADPSG	Fasting: ≤5.1 mmol/L; 1 h: ≤10.0 mmol/L; 2 h: ≤8.5 mmol/L	Any of the following PG values are exceeded: fasting: ≥5.1 mmol/L; 1 h: ≥10.0 mmol/L; 2 h: ≥8.5 mmol/L
